# Using formative research to enhance our understanding of implementation contexts: Preparing for a trial of maternal nutrition interventions

**DOI:** 10.1111/mcn.13639

**Published:** 2024-09-29

**Authors:** Andrew L. Thorne‐Lyman, Anna Kalbarczyk, Alison Tumilowicz, Parul Christian, Kaosar Afsana

**Affiliations:** ^1^ Department of International Health, Bloomberg School of Public Health Johns Hopkins University Baltimore Maryland USA; ^2^ Maternal, Newborn, and Child Health, Bill & Melinda Gates Foundation Seattle Washington USA; ^3^ Humanitarian Hub, James P. Grant School of Public Health BRAC University Dhaka Bangladesh

**Keywords:** balanced energy protein, Bangladesh, food supplement, formative, implementation research, micronutrients

## Abstract

- Formative research to understand the sociocultural and economic context into which interventions are introduced can help to maximize the uptake of interventions both in the context of effectiveness trials and ultimately for nutrition programs.
- Findings from the formative research study were used to inform different facets of the design of the effectiveness trial including providing evidence of the acceptability of the product, informing the decision to deliver the product to women's homes, and developing strategies to support adherence.

Traditionally, nutrition interventions during pregnancy in low‐ and middle‐income countries (LMICs) have received less attention than interventions focused on child nutrition. However, a solid body of evidence suggests that interventions to address micronutrient and macronutrient deficiencies during pregnancy could significantly improve both maternal and neonatal health along with foetal and child growth (Koivu et al., 2023; World Health Organization [WHO], 2016; Zavala et al., 2022) and need to be delivered at scale.

The WHO guidelines for a healthy pregnancy include several evidence‐based nutrition interventions. Along with iron‐folic acid (IFA), calcium and dietary education, the guidelines currently recommend multiple micronutrient supplementation (MMS) as a replacement for IFA ‘in the context of rigorous research’ and balanced energy and protein (BEP) supplementation specifically for contexts of high undernutrition (WHO, 2016).

The importance of finding ways to optimise the delivery of nutrition interventions to pregnant women has been highlighted in many recent reviews including the Lancet Series on Maternal and Child Nutrition (Heidkamp et al., 2021; Young & Ramakrishnan, 2020). A number of implementation research efforts are underway in countries throughout the world piloting MMS as a replacement for IFA implemented through antenatal care (ANC) (Horino et al., 2021; King et al., 2020). In contrast, it is less clear how BEP supplementation in pregnancy should be implemented as few examples of BEP supplementation programmes in pregnancy exist outside of emergency settings. This may be due to the high cost of food and the unique challenges of working with food commodities including storage and distribution.

The WHO specifically recommends a population‐based approach for BEP supplementation, focused on areas in which the population prevalence of low body‐mass index (<18.5 kg/m^2^) is greater than 20% (WHO, 2016). Only two countries (Bangladesh and India) meet this criteria at a national level (Christian et al., 2020), although many countries have subnational regions that exceed this prevalence (Victora et al., 2021). While WHO guidelines do not presently recommend the identification and supplementation of individual undernourished pregnant women, it has been argued that such an approach might optimise the benefits and costs of BEP supplementation (Christian et al., 2020).

Other than India, with its large national ICDS programme, Bangladesh is one of the few countries with experience implementing a large‐scale nutrition programme that included food supplementation of pregnant women. The Bangladesh Integrated Nutrition Programme (BINP) and National Nutrition Project (NNP), implemented from 1995 to 2006 was a large programme in rural Bangladesh that included supplementary feeding of pregnant women with a locally produced unfortified product, delivered through a network of community feeding centers staffed by women. Evaluations of this programme were mixed, however, and while there was evidence of impact in some subgroups, a number of implementation challenges were also identified, including ineffective targeting and sharing of supplementary food, low intensity, and the failure of behaviour change communication efforts to translate knowledge into practice (Nahar et al., 2009; White, 2005). One evaluation noted that ‘the programme might have been more successful if it had restricted its attention to the most malnourished women, improved targeting to reduce Type II error, and if it tried harder to discourage leakage and substitution’ (World Bank, 2005).

This collection of papers uses an implementation research lens to better understand the issues that could affect the implementation of a BEP intervention in the context of an effectiveness trial testing different targeting strategies for the delivery of BEP to pregnant women in a setting in Northwest Bangladesh. The rationale for implementing such a trial nearly two decades after the BINP/NNP ceased is grounded by several important realities. The first is that Bangladesh, like many countries, continues to have a high prevalence of low birth weight, which also contributes substantially to poor child growth (Mertens et al., 2023; Neupane et al., 2023). The second is that although BEP has been shown to be more effective in preventing small‐for‐gestational age births amongst undernourished populations (Ota et al., 2015), it is unclear what type of targeting approach will have the greatest impact on birth outcomes, or how to implement it in practice. The third is that consensus now exists around an improved formulation of a new generation of BEP (Bill & Melinda Gates Foundation [BMGF], 2017), which is fortified with 19 nutrients including 500 mg of calcium. A fortified ready‐to‐use, lipid‐based product made with puffed rice, lentil powder, milk solids and vegetable oils packed is currently being manufactured in Bangladesh with these specifications. The packaging of the product in 75 g sachets that are easy to store and distribute opens up new possibilities for programme implementation that could address some of the limitations related to the product used in the BINP/NNP.

The work presented in this collection of papers was developed recognising the need to adapt the interventions to be implemented during pregnancy, applying an untargeted versus targeted supplementation in a randomised effectiveness trial in northwest Bangladesh. We used qualitative research methods including key‐informant interviews and focus‐group discussions with married women, their husbands, mothers‐in‐law, and healthcare providers to inform the potential acceptability, delivery and use of the BEP supplement before launch of the trial. Topics of investigation were comprehensive, and included dietary beliefs in pregnancy, experiences of seeking and providing antenatal care, and perceptions of dietary supplements as an intervention to improve maternal and child health outcomes. A 2‐week test of the product was also conducted using the trials of improved practices method among a subset of women to evaluate acceptability, compliance and adherence related to the fortified BEP product to be used in the trial.

The design of the formative study and analysis and synthesis of the data were guided by an implementation science framework in nutrition comprising five domains (Tumilowicz et al., 2019). Figure 1 below depicts this framework and how the findings in each manuscript map to the domains.

**Figure 1:**
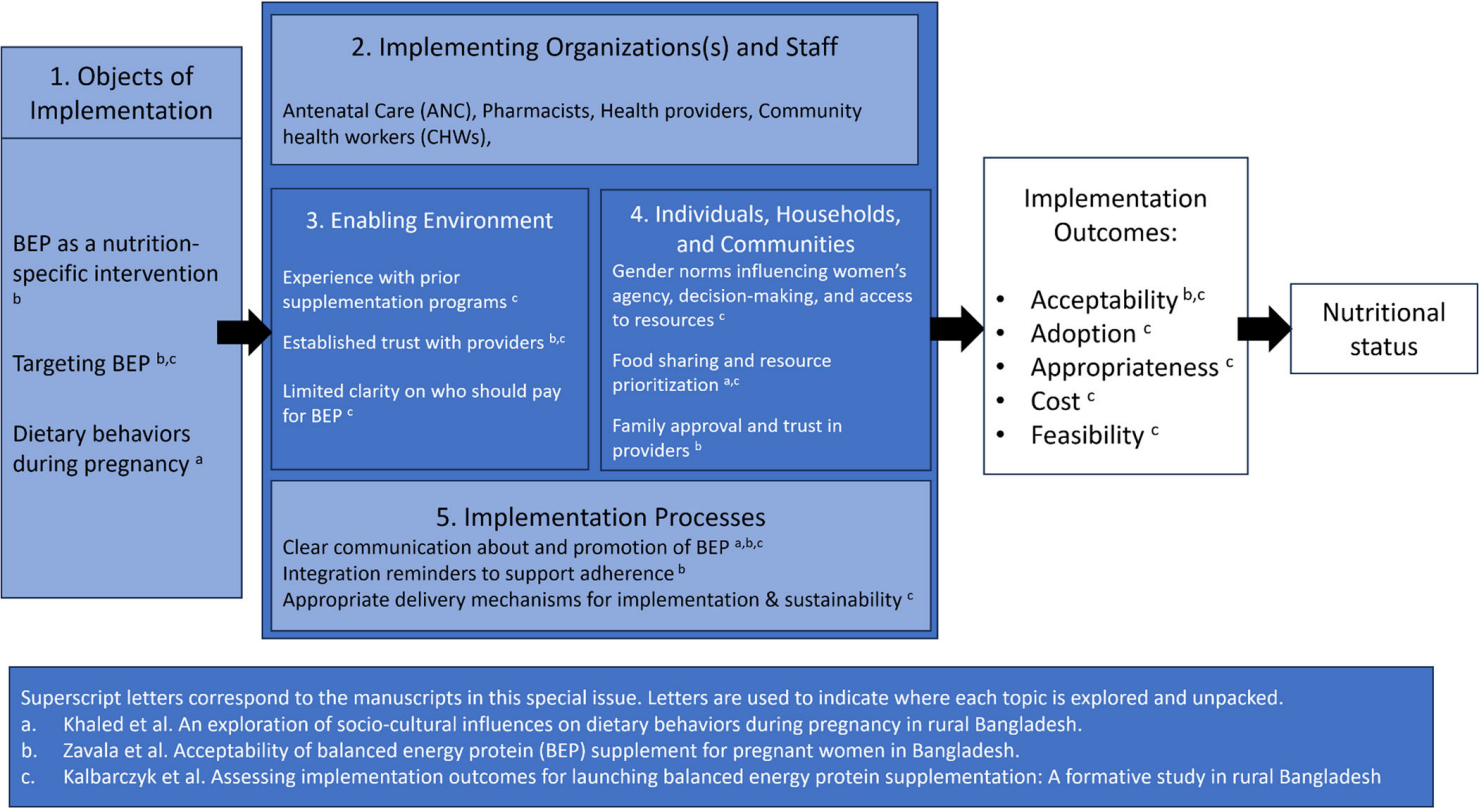
Key findings mapped to an implementation science framework in nutrition.

The first manuscript, by Khaled et al., explores modern perceptions of nutrition in pregnancy in the study setting (Khaled et al., 2024). It reveals that women had a clear concept of nutritious foods that aligned well with the nutrition education messages being provided through ANC in Bangladesh. Women also believed that the consumption of such foods in pregnancy benefitted the health of both women and their infants. At the same time, many pointed out the cost of such foods as a barrier to their consumption. The paper suggests that opportunities exist to build on these existing concepts, potentially framing the BEP supplement as a beneficial ‘add‐on’ that can support a healthy pregnancy.

The acceptability of the fortified BEP product is explored in the manuscript by Zavala et al. (2023). The BEP product was well accepted by women and providers who liked the taste and informative labelling. Compliance was high during a 2‐week test of the product and participants identified key strategies to support adherence including reminders from family members and/or providers and visual reminders in the home. Given their influencing and decision‐making roles, providers also suggested family‐oriented messaging to encourage household buy‐in.

The last paper, by Kalbarczyk et al., explores implementation outcomes (including acceptability, adoption, appropriateness, cost, and feasibility) related to BEP as an intervention and different distribution modalities (Kalbarczyk et al., 2023). In addition to being widely viewed as acceptable, BEP was also seen as an appropriate intervention for addressing undernutrition in pregnant women, particularly among the poorest segment of the population. Household access to financial resources and the cost of BEP were important concerns though there was no consensus whether BEP should be distributed for free versus at some cost. The issue of cost, and who should bear any costs, was linked to the feasibility of BEP as a future programme after the end of the trial. Participants noted that carefully crafted communication would be needed to promote adoption.

## 1 NEXT STEPS

The findings from the formative study presented in the three papers were used to inform the different facets of the design of the effectiveness trial (Clinicaltrials.gov ID NCT05576207) but may also be relevant to the ultimate implementation of future BEP programmes in Bangladesh. One of the key decisions to be made was how to best deliver the BEP intervention‐ at the home versus at a centralised point such as a market or healthcare provider. Ultimately home delivery was selected for the trial, informed by the finding that many women expressed that it would be difficult for them to regularly leave the home to pick up the product.

While gender barriers emerged in the research as an important consideration for women (i.e., freedom of movement and decision‐making), our research showed that these gender norms are changing. Assumptions we might have held about the present‐day role of mothers‐in‐law for example, were challenged in women's responses to our questions. Gender is a social construct which varies by context and can change over time (Lorber & Farrell, 1991). It is important to continue assessing gender norms, roles, and relations in formative research to account for such changes in intervention design and implementation.

Experience testing the product at home (Zavala et al., 2023) also highlighted the acceptability of the product, complementing positive findings from other feasibility and adherence studies of BEP products from Nepal including another ready to use paste (Lama, Khatry, et al., 2022; Lama, Moore, et al., 2022). Given the acceptance of BEP in this context, it is likely BEP would be well‐received by other populations and may help to enhance the effectiveness of other interventions that are co‐delivered, potentially including ANC itself as well as nutrition education.

The 2021 Lancet Series on Maternal and Child Nutrition stressed the need for more ‘how to’ research to find ways to deliver what is known to work (Shekar et al., 2021). Formative research to understand the sociocultural and economic context into which interventions are introduced, including in the design community‐based effectiveness trials can provide critical information that can help to maximise the uptake of interventions both in the context of effectiveness trials and ultimately for nutrition programmes (Tumilowicz et al., 2015).

## AUTHOR CONTRIBUTIONS

Andrew L. Thorne‐Lyman and Anna Kalbarczyk drafted the manuscript. All authors provided critical feedback and approved the final version.

## CONFLICT OF INTEREST STATEMENT

The authors declare no conflict of interest.

## Data Availability

Data sharing is not applicable to this article as no new data were created or analyzed in this study.
